# Comprehensive Analysis of RNA Expression and Isoform Diversity Reveals Molecular Mechanisms in Plant Responses to Diverse Thermal Conditions

**DOI:** 10.1007/s43657-025-00254-5

**Published:** 2026-06-24

**Authors:** Jianzheng Wang, Zesen Lai, Zhenze Lei, Fang Chang

**Affiliations:** 1 State Key Laboratory of Genetics and Development of Complex Phenotypes, Ministry of Education Key Laboratory of Biodiversity Sciences and Ecological Engineering and Institute of Biodiversity Sciences, Institute of Plant Biology, School of Life Sciences, Fudan University, Shanghai, 200438 China; 2 School of Tropical Agriculture and Forestry, Agriculture-Rural Affairs and Rural Revitalization, Hainan University, Haikou, 570228 China; 3 School of Biological Engineering, Enshi Polytechnic, Enshi, 445800 China

**Keywords:** Heat stress, Thermomorphogenesis, DNA damage response (DDR), Cell cycle regulation, Alternative splicing, Crop resilience

## Abstract

Elevated temperatures, including warm ambient temperature (WAT) and high ambient temperature (HAT), profoundly influence plant growth and development. While WAT promotes thermomorphogenesis and stimulates growth, HAT induces stress responses that can inhibit growth. Using *Arabidopsis thaliana*, we uncovered both shared and distinct molecular mechanisms underlying plant responses to these temperature conditions. We demonstrated that both WAT and HAT activate genes associated with the DNA damage response (DDR), but they differ in timing and intensity. While WAT encourages progression into the S phase of the cell cycle and enhances cell division, HAT induces G2/M arrest, indicating cell cycle inhibition caused by DNA damage accumulation and impaired DDR proteins. Notably, alternative splicing (AS), particularly intron retention (IR), emerged as a pivotal regulatory layer under heat stress. Prolonged high temperatures uniquely enriched IR events in genes involved in DNA repair and chromatin organization. Integrated analyses of AS and transcriptional changes revealed coordinated regulation, with IR events dominating genes that exhibit simultaneous upregulation. These findings highlight AS as an essential regulatory mechanism in plant responses to heat stress. Conserved responses to elevated temperatures in tobacco and tomato further suggest the presence of shared adaptive pathways across plant species. Our results provide novel insights into plant adaptation to fluctuating temperatures and highlight opportunities for engineering heat-resilient crops to mitigate the impacts of climate change.

**Supplementary Information:**

The online version contains supplementary material available at https://doi.org/10.1007/s43657-025-00254-5.

## Introduction

Global warming has increased the frequency of extreme weather and has posed substantial threats to the growth and development of all living organisms, including plants (Lesk et al. 2016; Lippmann et al. 2019; Wheeler and von Braun 2013). An increase of 1 ℃ in global mean temperature has been estimated to reduce the global yields of staple crops such as wheat, rice, maize, and soybean by 6.0%, 3.2%, 7.2%, and 3.1%, respectively (Zhao et al. 2017). Understanding how plants adapt and respond to elevated temperatures is therefore critical for developing strategies to mitigate the adverse effects of climate change and ensure global food security.

High ambient temperature (HAT) imposes heat stress (HS) on plants, leading to physiological and molecular disruptions. For instance, the model plant *Arabidopsis thaliana*, which thrives at an optimal temperature of ~ 22 °C, experiences severe stress at 34–38 °C, characterized by a global reactive oxygen species (ROS) burst, impaired photosynthesis, growth repression, cellular damage, rapid activation of molecular chaperones, and adjustments to cell membrane composition (Calleja-Cabrera et al. 2020; Hayes et al. 2021; Jacob et al. 2017; Kan et al. 2023; Liu et al. 2015; Ohama et al. 2017). Temperatures of 40 °C or higher can cause widespread damage, functional disruption, and eventual cell death (Vacca et al. 2004).

Despite these challenges, plants have evolved mechanisms to tolerate moderate temperature increases. Under warm ambient temperatures (WAT, 26–28 °C), *Arabidopsis* exhibits thermomorphogenesis, characterized by accelerated growth of the hypocotyl, primary root, petiole, and leaf, alongside a faster transition from vegetative to reproductive stages (Casal and Balasubramanian 2019; Kan et al. 2023; Lee et al. 2021; Lippmann et al. 2019; Yang et al. 2017). These morphological and architectural changes are accompanied by molecular adaptations, including the repression of growth/development-associated proteins and the selective expression of stress-responsive proteins and metabolites (Basu et al. 2016; Fahad et al. 2017; Kaplan et al. 2004; Lee et al. 2007; Weston et al. 2011; Yu et al. 2024).

Analyzing molecular phenotypic differences between WAT and HAT responses at a genome-wide level is essential for understanding how plants adapt to moderate heat while succumbing to extreme heat. It also offers valuable insights for molecular breeding of heat-tolerant crops. Recent studies have revealed certain distinct phenotypic and physiological responses caused by WAT and HAT in plants. While WAT generally promotes growth by enhancing cell elongation and differentiation, HAT inhibits growth by impairing cell division and photosynthesis. These divergent responses are regulated by distinct sets of transcription factors under different temperature regimes (González-García et al. 2023; Ibañez et al. 2017; Lippmann et al. 2019; Liu et al. 2022; Pedroza-Garcia et al. 2022; Quint et al. 2016). However, most studies have focused on specific genes or signaling pathways involved in the WAT or HAT response, leaving a gap in comprehensive genome-wide analyses of these processes.

Alternative splicing (AS), a post-transcriptional regulatory process where a single pre-mRNA is differentially spliced to produce multiple mRNA isoforms, has been implicated in heat stress responses across eukaryotes. Early studies in *Drosophila* revealed that heat stress-induced splicing inhibition contributes to developmental abnormalities under elevated temperatures (Yost and Lindquist 1986). Subsequent research demonstrated that heat stress-induced AS serves as a regulatory mechanism rather than a mere consequence of heat-induced damage in animals (Bond 1988; Gattoni et al. 1996; Shin et al. 2004; Shukla et al. 1990). In plants, numerous AS events have been identified under heat stress (Ding et al. 2014; Li et al. 2020; Lin and Zhu 2021; Ling et al. 2021), further highlighting their importance in heat stress adaptation. However, despite these insights, the role of AS in response to elevated temperatures remains inadequately explored, particularly at the genome-wide level in plants.

In this study, we conducted a comprehensive analysis using *Arabidopsis thaliana* to investigate the molecular responses to elevated temperatures. By integrating transcriptome profiling and alternative splicing analysis, we identified both common and temperature-specific differentially expressed genes (DEGs) in response to warm ambient temperature (WAT, 28 °C) and high ambient temperature (HAT, 37 °C) conditions, in comparison to normal growth condition (22 °C). Importantly, we observed that these temperature-responsive mechanisms are conserved across different species, including tobacco (*Nicotiana benthamiana*) and tomato (*Solanum lycopersicum*), underscoring the broad relevance of our findings.

## Materials and Methods

### Plant Materials and Growth Conditions

*Arabidopsis thaliana* used in this study was of the Columbia (Col-0) ecotype. The seeds were surface-sterilized in ethanol mixture (ethanol: ddH_2_O, 7:3) for 10 min, then germinated on solid agar plates containing Murashige and Skoog (MS) medium (2.2 g/L MS, 5 g/L sucrose, and 4.5 g/L phytagel [pH 5.8]). All seedlings were grown under long-day conditions (16 h light, 8 h dark). For phenotyping, seedlings were grown at 22 °C for four days before being transferred to 28–37 °C conditions. For RNA-seq, seedlings were grown vertically on MS medium at 22 °C for seven days or for eight days and 22 h before the treatments began. Tobacco and tomato seedlings were grown under long-day conditions (16 h light, 8 h dark). Tobacco seedlings were initially grown vertically on MS medium at 22 °C for four days, followed by a transfer to 28–37 °C conditions for an additional three days. Tomato seeds were germinated in distilled water for two days and then grown in seed trays with Hoagland nutrient solution at 22 °C for four days before being transferred to 28–37 °C for an additional three days.

### RNA Extraction and Sequencing

Seven-day-old Col-0 seedlings subjected to various treatments were harvested at 3:00 PM (seven hours after the onset of the light cycle). Total RNA was extracted from the seedlings using TRIzol reagent (Invitrogen) following the manufacturer’s protocol. The assessment of RNA quality, library construction, and sequencing were conducted by BioMarker (Qingdao, China). RNA integrity was assessed using an Agilent Bioanalyzer, and samples with an RNA integrity number (RIN) greater than 7 were selected for library preparation.

For mRNA-seq library preparation, mRNA was isolated using Oligo(dT)-attached magnetic beads and subsequently fragmented in fragmentation buffer. The fragmented mRNA was then used as a template for first-strand cDNA synthesis with random hexamer primers. Second-strand cDNA was subsequently synthesized by adding PCR buffer, dNTPs, RNase H, and DNA polymerase I. Following purification with AMPure XP beads, the double-stranded cDNA was subjected to end repair, 3′ end adenylation, and adapter ligation. AMPure XP beads were then used to select fragments of the desired size range. Finally, the unstranded cDNA libraries were amplified via PCR using the following adapters: 3'-AGATCGGAAGAGCACACGTCTGAACTCCAGTCAC and 5'-AGATCGGAAGAGCGTCGTGTAGGGAAAGAGTGT. The qualified libraries were then sequenced on an Illumina NovaSeq 6000 platform using paired-end 150 bp (PE150) reads.

### Transcriptome Data Analyses

The RNA-seq processing was performed as previously described (Lai et al. 2022). Briefly, the raw reads were trimmed to remove adapter sequences using Trim-galore (https://github.com/FelixKrueger/TrimGalore/). The trimmed reads were mapped to the TAIR10 using STAR with default settings (Dobin et al. 2013). Based on the TAIR 10 annotation, reads mapped to unique positions were counted by featureCounts (Liao et al. 2014). RNA-seq count data were processed using the edgeR package (Robinson et al. 2010). The data matrix was filtered to retain genes with a CPM (counts per million) value greater than one in at least two samples. Normalization factors were calculated using the TMM (Trimmed Mean of M-values) method. Generalized linear models (GLMs) were fitted, and differential expression was assessed using the likelihood ratio test (LRT). The final list of DEGs was determined with an absolute fold change ≥ 2 (treatment vs. control) and an FDR ≤ 0.05.

### GO Analysis

The biological function enrichment of selected gene sets was performed using clusterProfiler 4.0 (Wu et al. 2021), with the criterion *q-value* was ≤ 0.05.

### Real-Time qPCR and RT-PCR

Total RNA extraction of seedling/root tips was performed with TRIzol (TaKaRa RNAiso Plus), and DNA Eraser Set (TaKaRa) was used to remove DNA. For each sample, 1 µg of total RNA was used for cDNA synthesis with the reverse transcription system (TaKaRa RR047A). The cDNA reaction mixture was diluted to 100 µL. The qPCR reaction was performed with TB Green^®^ Premix Ex TaqTM II (Tli RnaseH Plus) (TaKaRa RR820A) in a Bio-Rad CFX96 Touch. *AT1G13320* was used as a control for expression level normalization. The primers used for real-time PCR analysis are listed in Table S15.

RNA extraction, reverse transcription (oligo(dT) primer), and PCR cycling (35 cycles) were performed following RNA-seq experimental conditions, with primers listed in Table S15.

### GUS Staining

Seedlings of *CYCB1;1::GUS* reporter line (Colón-Carmona et al. 1999) or *DR5::GUS* lines were subjected to different treatment conditions. Then the seedlings were submerged in GUS staining solution and incubated 3 h in the dark at 37 °C. The GUS staining pictures were taken using ECLIPSE Ni-U (Nikon).

### EdU Assay

The EdU staining was conducted using a BeyoClick EdU Cell Proliferation Kit with Alexa Fluor 488 (Beyotime) according to the manufacturer's instructions, and images were taken on a confocal microscope (FV3000, Olympus). Briefly, five-day-old seedlings treated under different conditions were transferred to 20 µM EdU-containing liquid MS medium and incubated at room temperature for two h. The seedlings were then fixed in 4% (w/v) paraformaldehyde for 15 min. After being washed three times with 1×PBS, the seedlings were incubated in 0.3% Triton X-100 for 15 min. Following two washes with 1×PBS, the seedlings were incubated in the Click Additive Solution for 30 min in the dark. Finally, the solution was removed, and the seedlings were washed twice with 1×PBS. Images were acquired using a confocal microscope (FV3000, Olympus) with an excitation wavelength of 488 nm and an emission wavelength range of 491–550 nm.

### Alternative Splicing Analysis

For the alternative splicing analysis, the alignment files generated by STAR and the annotation files from AtRTD3 (Zhang et al. 2022) were used as input for rMATS (v4.1.2) using the Junction Counts (JC) method to test for differentially spliced transcripts. Alternative splicing events were identified by rMATS (v4.1.2), and sites with a *p-value* < 0.05 between the compared pairs were considered differentially spliced sites.

RT-PCR was used to validate the randomly selected differential alternative splicing (DAS) events. Specifically, seven intron retention (IR) events were randomly chosen from IR-type DASGs that displayed high transcript abundance in our RNA-seq data (transcripts per million (TPM) > 1 in at least two experimental samples, totaling 3,354 genes). Additionally, four DAS events were randomly selected from the 56 DNA repair-related DASGs identified in the 48 h HAT group.

## Results

### Transcriptomic Responses to Heat Stress Vary Depending on the Degree of Elevated Temperatures

**Fig. 1:**
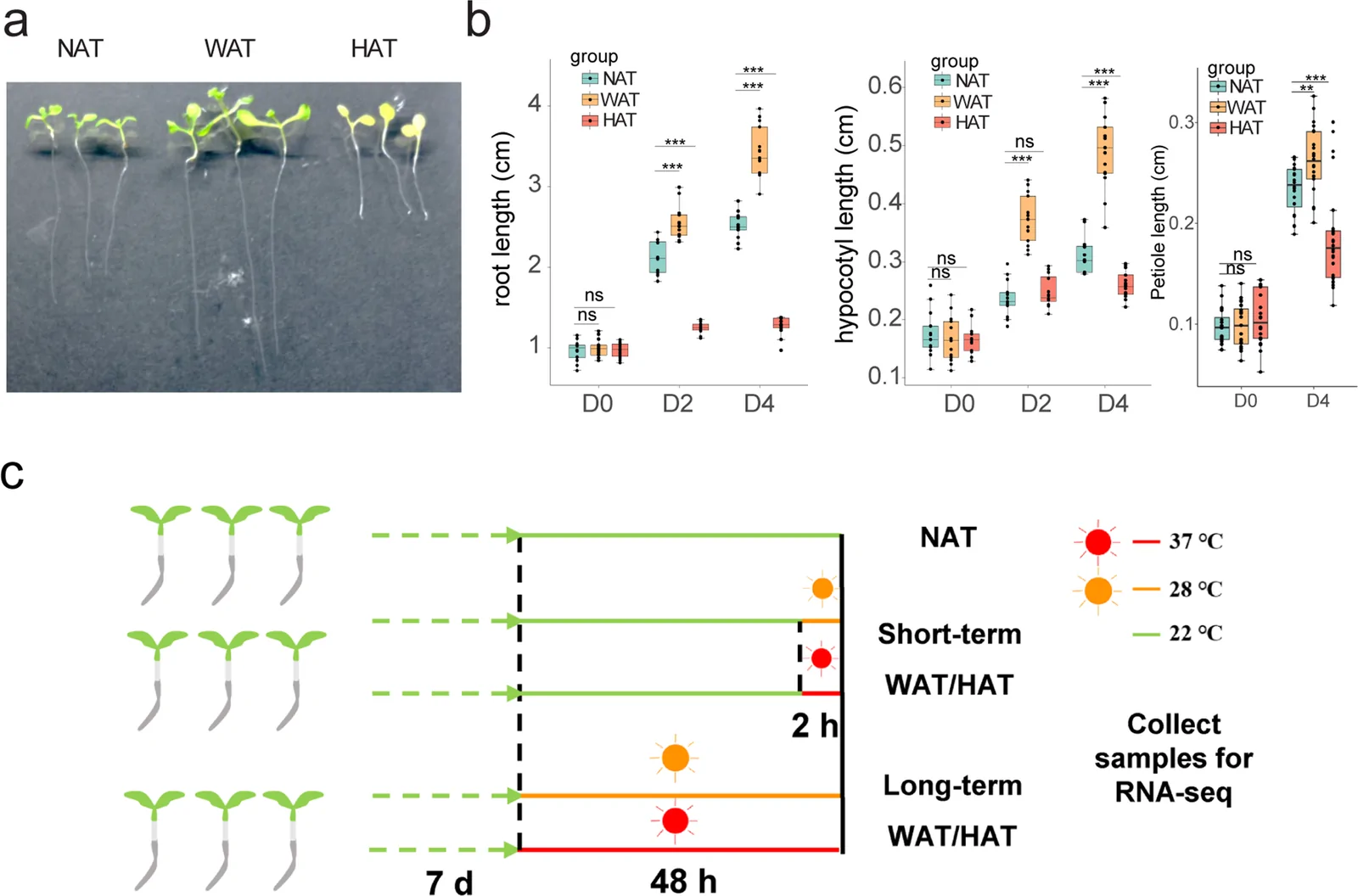
Plant developmental responses under different heat stress conditions. **a** Phenotype of 8-day-old seedlings initially grown at NAT for four days, followed by a transfer to WAT or HAT conditions for an additional four days. **b** Quantitative data on primary root length, hypocotyl length, and petiole length of seedlings from day zero to day four after transfer to treatment conditions are presented as mean ± s.d. (*n* ≥ 10). **c** Flowchart depicting the RNA-seq analysis for two elevated temperature treatments. RNA samples were collected from WT seedlings grown at 22 °C for seven days, followed by transfer to either 28 °C (WAT) or 37 °C (HAT) for two days (48 h) or 2 h, respectively. Statistical analysis was performed using Student’s *t*-test: *, *p* < 0.05; **, *p* < 0.01; ***, *p* < 0.001, ns, not significant

To assess how plants respond to different levels of elevated temperature, we first evaluated the differential effects of WAT and HAT on plant growth. Wild-type *Arabidopsis* seeds were initially grown at normal ambient temperature (NAT, 22 °C) for four days. The seedlings were then transferred to either WAT (28 °C) or HAT (37 °C) conditions for an additional four days. Afterward, primary root length, hypocotyl length, and petiole length were measured and compared (Fig. 1a, b). As expected, root growth was significantly inhibited under HAT, whereas it was promoted under WAT. The growth of the hypocotyl and petiole was also enhanced under WAT but inhibited under HAT (Fig. 1a, b), consistent with recent studies (Ai et al. 2023; González-García et al. 2023; Yang et al. 2017).

To further elucidate the molecular phenotypes underlying these distinct responses, we performed whole-genome RNA sequencing (RNA-seq) on *Arabidopsis* seedlings exposed to WAT or HAT for 2 h and 48 h, respectively. Seedlings grown under NAT at the same developmental stage were used as controls (Fig. 1c). Three biological replicates were performed for each sample, each achieving more than 20 million mapped reads with high correlation coefficients between biological replicates (Table S1, Table S2, R^2^ > 0.96). The expression of a total of 20,302 genes was detected by RNA-seq (Table S3). Principal component analysis (PCA) and hierarchical clustering analysis demonstrated that biological replicates clustered together (Fig. S1a, b). For each treatment group, randomly selected genes were analyzed using reverse transcription quantitative PCR (RT-qPCR) for validation. The results showed a strong correlation with the RNA-seq data (Fig. S1c, R^2^ ≥ 0.8), confirming the reliability of our RNA-seq findings.

### Elevated Temperatures Induce Both Similar and Distinct Gene Expression Changes

**Fig. 2:**
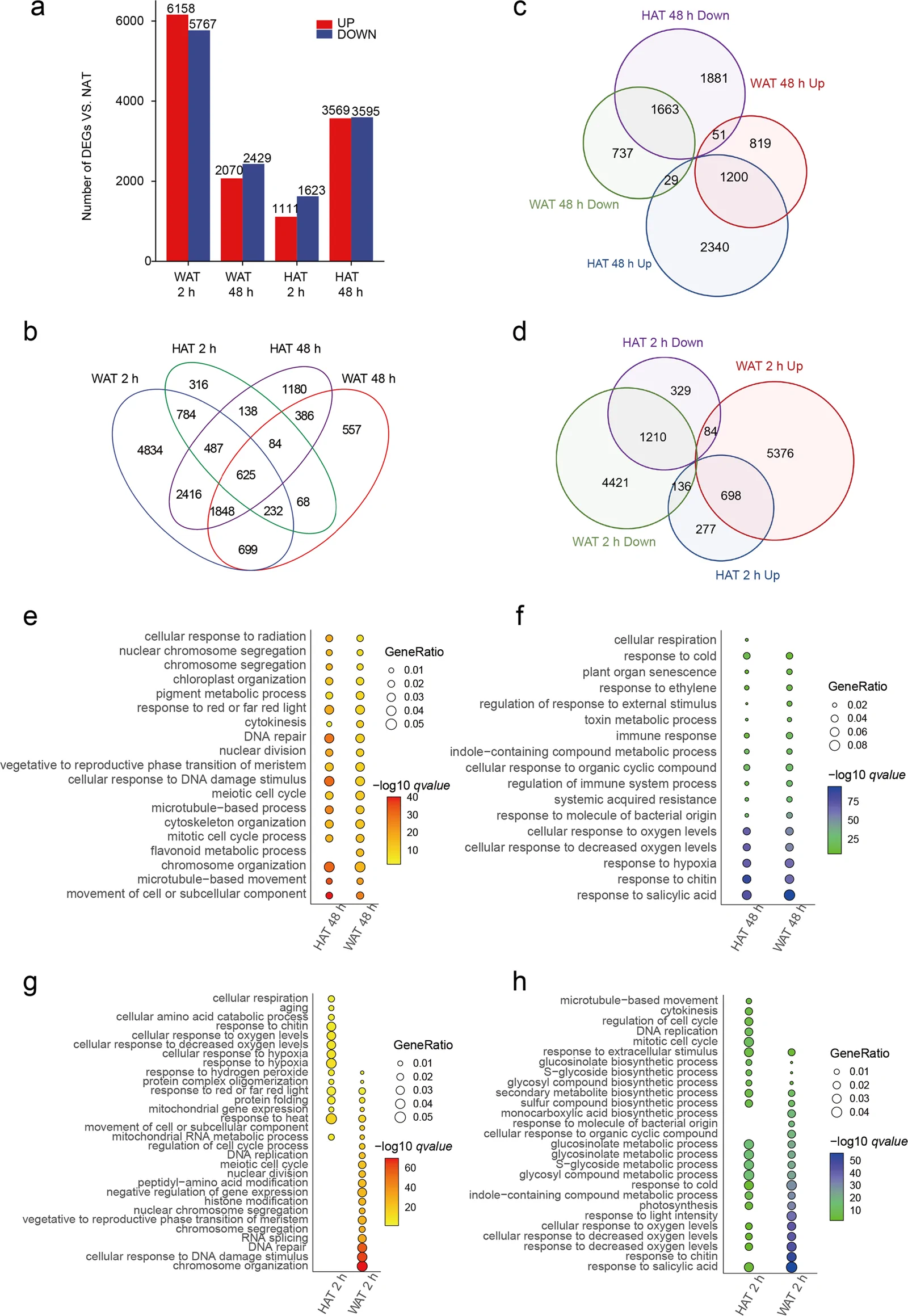
Effects of WAT and HAT treatments on *Arabidopsis* seedlings. **a** Number of DEGs after 2 h or 48 h of WAT or HAT treatments. Red bars represent up-regulated genes, while blue bars represent down-regulated genes. **b** Venn diagram showing the overlap of DEGs among the treatment groups. **c** Venn diagram howing the overlap of DEGs in the long-term treatment groups. **d** Venn diagram howing the overlap of DEGs in the short-term treatment groups. **e-h** Dot plots comparing Gene Ontology (GO) analysis of up-regulated genes (e, g) and down-regulated genes (f, h)

Under HAT conditions for 2 h, we identified 2734 differentially expressed genes (DEGs), including 1111 up-regulated and 1623 down-regulated genes. After 48 h of HAT exposure, the number of DEGs increased to 7164, with 3569 up-regulated and 3595 down-regulated genes. In contrast, under WAT conditions, a markedly different gene expression profile was observed: 11,926 DEGs were identified after 2 h of WAT exposure, with 6158 up-regulated and 5767 down-regulated genes, suggesting that short-term (2 h) WAT induces a more immediate and intense transcriptional response. After 48 h, the number of DEGs decreased to 4499, consisting of 2070 up-regulated and 2429 down-regulated genes (Fig. 2a, Table S3).

The consistency and reproducibility of these gene expression patterns were validated by comparing our results with those from previous studies, which showed significant overlap: 79.5% with Qin et al. (2022) (7-day-old seedlings, 28 °C, 3-day treatment), 75.7% with Jin et al. (2020) (7-day-old seedlings, 28 °C, 4-day treatment), and 68.1% with Xue et al. (2021) (7-day-old seedlings, 27 °C, 12 h light treatment) for WAT DEGs. Similarly, for HAT DEGs, we found overlaps of 47.9% with Han et al. (2021) (7-day-old seedlings, 37 °C, 30 min treatment) and 40.6% with Blair et al. (2019) (13-day-old seedlings, 37 °C, 1 h treatment; Fig. S1d, e). Despite differences in treatment conditions, photoperiods, and growth stages, the significant overlap in DEGs across these studies underscores the robustness and reliability of our RNA-seq data. By comparing the expression levels of genes across all four treatment groups relative to those at 22 °C conditions, we identified 625 genes that were commonly affected in all groups (Fig. 2b). These 625 DEGs were categorized into three clusters (Fig. S2a, Table S4): Cluster 1 (159/625), which shows significant up-regulation; Cluster 2 (366/625), which exhibits significant down-regulation; and Cluster 3 (100/625), which showed differential expression patterns under WAT and HAT treatments. In Cluster 1, consisting of 159 genes, we identified nine heat shock protein (HSP) genes, including *HSP17.4A*, *HSP17.6*, *HSP18.1*, and *HSP90-5*. The expression of HSP genes is a hallmark of the heat shock response, with heat stress transcription factors (HSFs) playing crucial regulatory roles in this process (Jacob et al. 2017). We identified 59 HSP or HSF genes with altered expression in at least one treatment group (Table S5), with higher levels observed in short-term treatments compared to long-term treatments (Fig. S2b). Notably, *HsfA2* expression initially increased under short-term heat shock but rapidly declined with prolonged stress, consistent with previous reports (Fig. S2c) (Li et al. 2005; Nishizawa et al. 2006). Additionally, we identified *ABF3*, involved in ABA signaling, and *ABA3*, essential for ABA biosynthesis (Ghassemian et al. 2000; Jin et al. 2023), as well as *CCA1*, *ARF11*, and*SR30* in Cluster 1. Recent studies have indicated that elevated ambient temperatures can significantly increase pathogen virulence. Within Cluster 2, we identified several immune and salicylic acid (SA) response genes, including *NIMIN1*,* NIMIN2*,* PCC1*,* ACD6*,* LURP1*, and *PCR1* (Fig. S2c). The presence of these common DEGs, particularly those consistently changing in the same direction, suggests that plants may activate shared pathways to cope with elevated temperatures.

### Distinct and Shared Pathways in Long-Term WAT and HAT Responses

To gain insights into the biological processes in response to long-term and short-term elevated temperature conditions, we performed Gene Ontology (GO) analysis of biological process (Table S6-S13). Initially, we assessed the responses to long-term treatments, which induced various morphological changes. Among the 8720 DEGs identified in the long-term treatment groups, 27.03% (1200/4439) were commonly up-regulated, while 38.13% (1663/4361) were commonly down-regulated under both WAT and HAT treatment conditions (Fig. 2c).

GO analysis of the long-term treatment groups revealed that a total of 338 GO terms (39.9%, 338/868) were significantly enriched in both the WAT and HAT groups. Among these, 218 GO terms were associated with up-regulated DEGs, while 120 GO terms were associated with down-regulated DEGs (Fig. S3a). Notably, the common GO terms for up-regulated DEGs included microtubule-based movement, chromosome organization, mitotic cell cycle process, indicating that both WAT and HAT condition for 48 haffect the cell cycle process (Fig. 2e; Fig. S3b). Conversely, common GO terms for down-regulated DEGs at 48 h included response to salicylic acid, response to chitin, and response to hypoxia (Fig. 2f; Fig. S3f). This significant overlap in GO terms highlights the shared biological processes impacted by both treatment conditions, underscoring their similar effects on plant responses.

To identify the differences between long-term WAT and HAT responses, we examined specific GO terms in the 48 h WAT or HAT treatment groups. Genes related to mRNA processing and RNA splicing were specifically up-regulated in the HAT 48 h group (e.g., *AT1G09140*,* AT4G02430*, *and AT1G60200*), suggesting that pre-mRNA splicing plays a role in the heat stress response in seedlings (Fig. S3c). This finding aligns with previous reports of heat stress affecting pre-mRNA splicing in various eukaryotes, in which accurate alternative splicing is crucial for a range of plant physiological responses (Ling et al. 2021). In contrast, the WAT 48 h group exhibited specific enrichment in GO terms related to flavonoid metabolism, cell wall biogenesis, S-glycoside metabolism, and plant-type cell wall biogenesis (Fig. S3d). The GO enrichment profiles of down-regulated DEGs also revealed distinct differences between the WAT 48-hour and HAT 48-hour groups (Fig. S3e). The HAT 48-hour group showed significant enrichment in processes related to the generation of precursor metabolites and energy, and cellular respiration (Fig. S3g). Conversely, the WAT 48 h group displayed enrichment in intracellular signal transduction and cellular response to nitrogen levels (Fig. S3h). Overall, these analyses highlight how long-term elevated temperature treatments uniquely regulate genes involved in stress responses, development, pigment metabolism, cell cycle, and DNA repair.

### Short-Term WAT and HAT Treatments Induce Distinct Effects on Gene Expression Profiles

To gain a deeper understanding of the differences and similarities in seedlings' responses to WAT and HAT conditions, we inspected the molecular phenotype of seedlings under short-term temperature treatments. We identified 698 commonly up-regulated DEGs and 1210 commonly down-regulated DEGs under short-term HAT. In the WAT 2 h group, 647 GO terms were significantly enriched among the up-regulated DEGs, compared to 57 in the HAT 2 h group (Fig. 2d; Fig. S4a). Furthermore, we identified 17 GO terms enriched in both WAT and HAT groups, indicating shared molecular processes activated by both treatments (Fig. S4a). These commonly up-regulated GO terms were primarily associated with RNA modification, response to heat, mitochondrial RNA processing, and protein folding (Fig. 2g; Fig. S4b).

In contrast, down-regulated DEGs exhibited 69 enriched GO terms in both groups, with 274 specifically enriched in the WAT 2 h group and 73 in the HAT 2h group (Fig. S4e). Common GO terms in the down-regulated DEGs included response to salicylic acid, response to hypoxia, photosynthesis, and response to cold. Up-regulated DEGs in the WAT 2 h group were specifically enriched in GO terms associated with DNA repair, RNA splicing, histone modification, and DNA replication (Fig. 2h; Fig. S4c). Conversely, down-regulated DEGs in the HAT 2 h group were enriched in GO terms related to the mitotic cell cycle, cytokinesis, and DNA replication (Fig. S4g). These contrasting effects on genes related to the mitotic cell cycle suggest distinct impacts on cell cycle progression. Overall, these findings underscore the unique effects of short-term WAT and HAT conditions on various biological processes, highlighting specific molecular responses to varying levels of elevated temperature.

### DNA Damage Response-Related Genes Are Induced Under Elevated Temperatures in *Arabidopsis* Seedlings

**Fig. 3:**
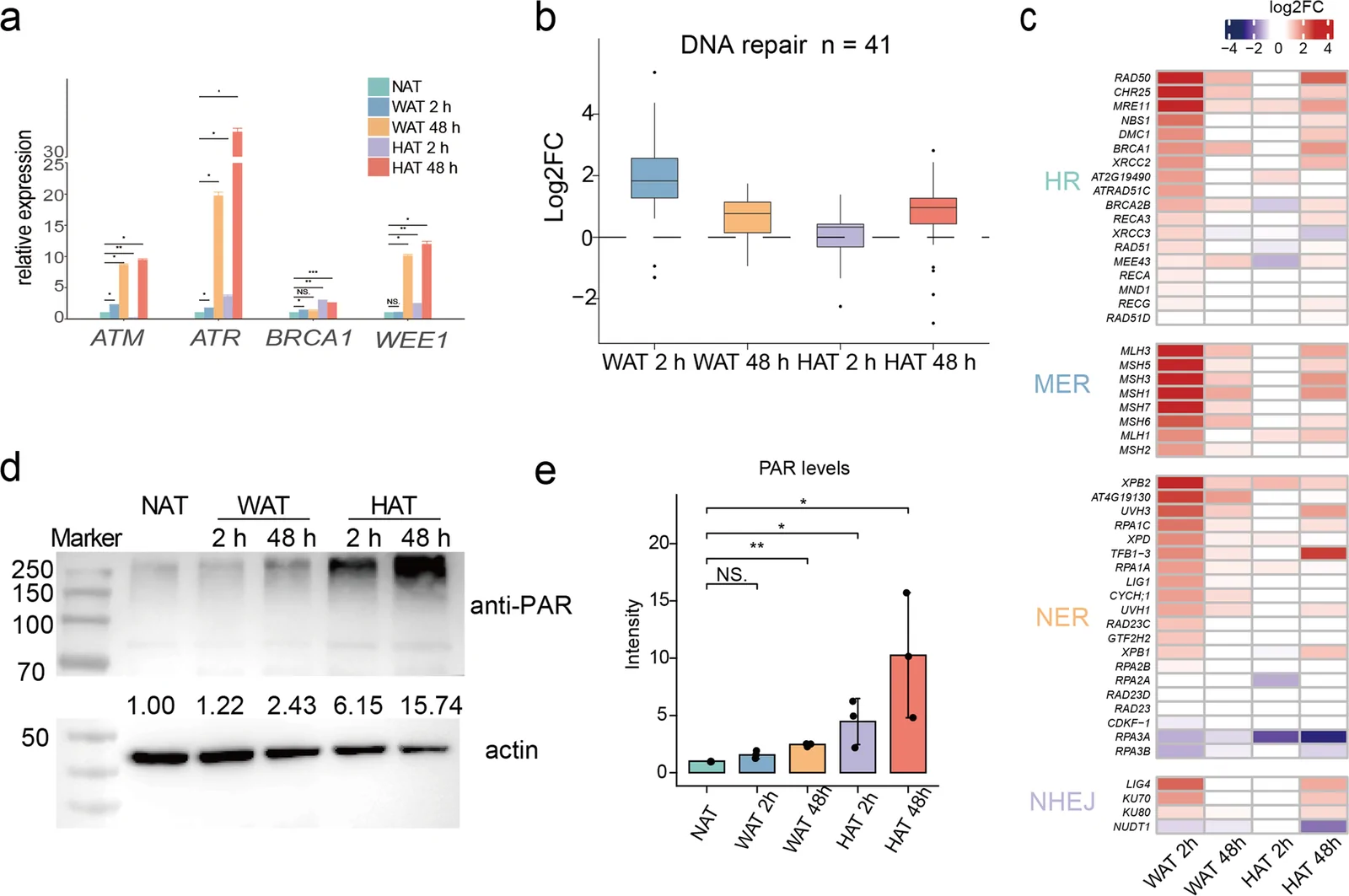
WAT and HAT activate genes involved in DNA damage response (DDR) with differing timing and intensity. **a** RT-qPCR analysis of *ATM*,* ATR*,* BRCA1,* and *WEE1* expression under WAT and HAT. **b** A boxplot shows the expression levels of DEGs that are involved in DNA repair. **c** A heatmap displays the expression levels of four types of DNA repair-related genes. **d**, **e** Detection of poly ADP-ribosylation levels under WAT and HAT using an anti-PAR antibody. Actin served as a loading control. Three biological replicates were performed. Statistical analysis method was performed using Student’s t-test: *, *p* < 0.05; **, *p* < 0.01; ***, *p* < 0.001, ns, not significant

It has been reported that high temperature stress causes various types of DNA damage and impedes DNA repair (Han et al. 2020; Hu et al. 2016; Kantidze et al. 2016). Plants possess a complex DNA damage response (DDR) mechanism to confront DNA damage, including several highly conserved elements (Pedroza-Garcia et al. 2022). Members of the phosphatidylinositol 3-kinase-like family, ATM and ATR (Rad3-related), are primarily activated by DNA double-strand breaks (DSBs) and single-stranded DNA (ssDNA) or defects in replication fork progression (Maréchal & Zou 2013). We observed significant induction of *ATR* expression in all four groups (Fig. 3a). Additionally, we noted an increase in *ATM* expression following the WAT treatment and long-term HAT conditions, but a slight decrease after short-term HAT treatment (Fig. 3a). Interestingly, except for the HAT 2 h group, GO terms related to DNA repair were significantly enriched among the DEGs (Fig. 2e, h).

To investigate the effects of different durations and levels of high temperature, we analyzed the global expression patterns of DNA repair-related genes under both WAT and HAT treatment conditions (Takahashi et al. 2019). The transcript levels of 41 DNA repair-related genes significantly changed under at least one of the four treatments (Fig. 3b). Most genes associated with DNA repair exhibited increased expression levels in the WAT 2 h, WAT 48 h, and HAT 48 h groups, especially in WAT 2 h (Fig. 3b). In plants, key DNA repair pathways include homologous recombination (HR), non-homologous end-joining (NHEJ), mismatch excision repair (MER), and nucleotide excision repair (NER) (Manova and Gruszka 2015). We identified 13 differentially expressed genes (DEGs) involved in HR, with some consistently showing up-regulation across both WAT and HAT groups (Fig. S5a). Similarly, MER- and NER-related genes exhibited up-regulation in the WAT 2 h group and increased expression in both the WAT 48 h and HAT 48 h groups (Fig. S5b, S5c, Table S14). Additionally, NHEJ-related genes were up-regulated in the WAT 2 h and HAT 48 h groups (Fig. 3c, Table S14). Overall, our findings suggest that elevated temperatures modulate the expression of genes associated with all four types of DNA repair mechanisms. Notably, 36 out of 50 DNA repair-related genes exhibited up-regulation in the WAT 2 hgroup, with 13 genes showing consistent differential expression in both the WAT 2 h and WAT 48 h groups, indicating potential activation of DNA repair mechanisms under non-stressful elevated temperatures (Fig. 3c). Conversely, the HAT 2 hgroup displayed fewer changes in DNA repair-related genes, whereas the HAT 48 hgroup exhibited differential expression in 18 of the 50 genes (Fig. 3c), suggesting that DNA repair-related genes are activated in both WAT and HAT conditions, but with distinct temporal patterns.

Protein poly(ADP-ribosylation) (PARylation), catalyzed by poly(ADP-ribose) polymerases (PARPs), is a crucial post-translational modification (PTM) involved in the DNA damage response. PARylation plays a key role in base excision repair, single-strand break (SSB) repair, and double-strand break (DSB) repair, by facilitating the recruitment of DNA repair proteins to damage sites and stabilizing repair complexes (Gu et al. 2019; Liu et al. 2017). To evaluate the activation of DNA damage response (DDR), we detected the levels of PAR in seedlings exposed to WAT and HAT conditions. After 2 hor 48 hexposure to WAT and HAT, respectively, PARP activity levels increased compared to the control group (NAT) (Fig. 3d-e). Notably, the level of PARP activity was much higher in both short-term and long-term HAT conditions than in the WAT conditions. In addition, the 48 hHAT condition induced much higher PARP activity compared to 2 hHAT exposure (Fig. 3d, e), suggesting more severe DNA damage in prolonged HAT conditions. Collectively, these results suggest that DNA damage may be induced by both WAT and HAT, and could accumulate under HAT conditions.

### Elevated Temperatures Alter the Cell Cycle Progression in Root Tips of *Arabidopsis*

**Fig. 4:**
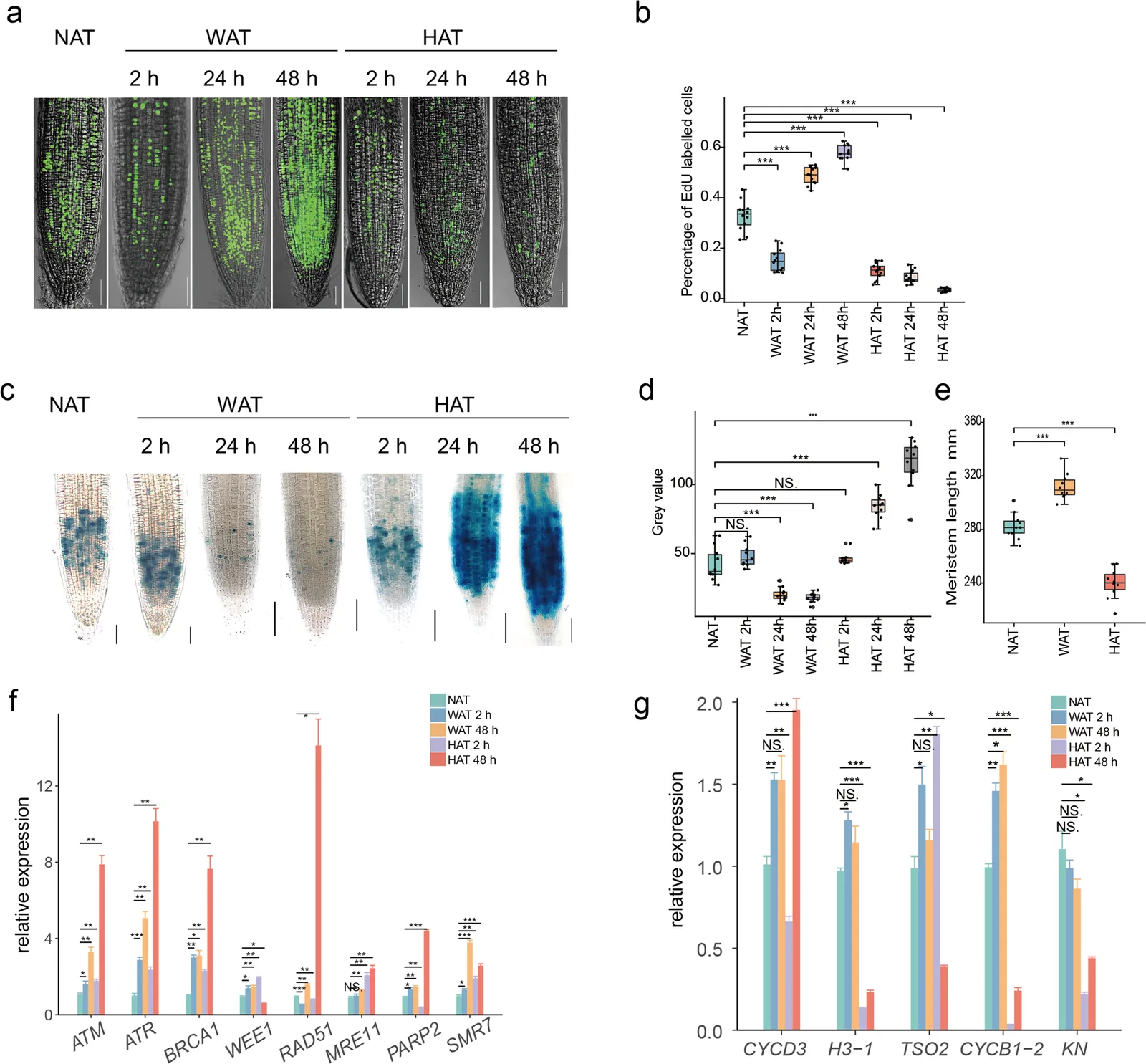
WAT enhances cell division, while HAT induces DNA damage accumulation and G2/M arrest in root tips. **a** S-phase entry of mitotic cells in Col-0 roots was visualized by EdU staining. **b** Quantification of EdU-labeled cells in the root meristem. **c** GUS staining of *CYCB1;1::GUS* under NAT, WAT, and HAT conditions. **d** Quantification of *CYCB1;1::GUS* activity in root tips (*n* > 10). **e** Quantification of root meristem size (*n* > 10). **f** RT-qPCR analysis of DDR-related genes in root tips in response to elevated temperatures. **g** RT-qPCR analysis of cell cycle-related genes in root tips in response to elevated temperatures. Five-day-old WT seedlings were subjected to 2 or 48 h of WAT or HAT treatment, and root tips were collected for total RNA extraction and RT-qPCR analysis. Statistical analysis was performed using Student’s *t*-test: *, *p* < 0.05; **, *p* < 0.01; ***, *p* < 0.001, ns, not significant

Cell cycle checkpoint control mechanisms, as components of the DNA damage response (DDR), can pause the cell cycle to facilitate DNA repair or trigger cell differentiation or programmed cell death to prevent the propagation of DNA errors during mitosis or meiosis (Pedroza-Garcia et al. 2022). Our findings suggest that genes associated with the DNA repair system are likely induced by both WAT and HAT, indicating the activation of the DDR and potential alterations in cell cycle progression. Previous research has demonstrated that heat stress (37 °C) can inhibit root growth and cause G2 arrest through Rep-MYB accumulation in an ANAC044/ANAC085-dependent manner in *Arabidopsis* primary roots (Takahashi et al. 2019). To investigate the impact of different temperature treatments on the cell cycle, we utilized 5-ethynyl-2’-deoxyuridine (EdU) to label root cells that had entered the cell-cycle S phase and were undergoing DNA replication (Kotogány et al. 2010). At the 2 h mark following the transition from NAT to WAT, there was a slight decrease in the number of Edu-labeled cells. However, after 24 h or 48h of WAT treatment, a significant increase in the number of EdU-labeled cells was observed compared to the NAT control (Fig. 4a, b). This suggests that prolonged WAT treatment promotes the entry of cells into the S phase of the cell cycle and enhances cell division activity in the root tips. In contrast, under HAT conditions, the number of Edu-labeled cells was dramatically lower at all three time points compared to the control group, indicating that HAT treatment considerably inhibited cell division in root tips (Fig. 4a, b). Additionally, we examined the expression of the *CYCB1;1::GUS* reporter system in root tips. Similarly, the late G2/M marker *CYCB1;1::GUS* signal significantly increased after both short-term and long-term HAT treatments. Under WAT conditions, *CYCB1;1::GUS* signal exhibited a slight increase at 2h , followed by a significant decrease at 24 hand 48 h (Fig. 4c, d). Moreover, we observed that the meristem length of the WAT 48 h group was longer than that of NAT group, while the HAT 48 hgroup exhibited a dramatic reduction in meristem length compared to NAT group (Fig. 4e). It has been reported that *CYCB1;1* expression is strongly induced in cells experiencing DNA damage (Schnittger and De Veylder 2018). Taken together, these findings suggest that heat stress induces DNA damage, leading to cell cycle arrest. In contrast, prolonged exposure to warm ambient temperatures accelerates the entry into the S phase of the cell cycle in *Arabidopsis* seedlings.

Furthermore, considering the close association between cell division and auxin, we evaluated the activity of the *DR5::GUS* marker under both WAT and HAT conditions. We observed a slight increase in GUS activity under WAT conditions, while a significant increase in GUS activity was observed under HAT conditions (Fig. S6a, b). These data indicate that auxin signaling in root tips is influenced by both WAT and HAT. Consistently, previous studies have reported that both exogenous auxin application and high temperature enhance the inhibition of root growth (González-García et al. 2023). Additionally, it has been observed that under WAT conditions, there is an increase in auxin levels, which in turn promotes primary root elongation (Ai et al. 2023). Taken together, these findings suggest that elevated temperatures induce changes in auxin levels and alter cell division rates in the root apical meristem. This, in turn, might lead to enhanced primary root length under WAT conditions and inhibited root growth under HAT conditions.

To further explore gene expression changes related to DDR and cell cycle progression under WAT and HAT conditions, we focused on the actively dividing root tips of seedlings. We collected root tips from seedlings subjected to short-term and long-term WAT/HAT treatments, using NAT conditions as the control, to evaluate the impact of temperature on DDR and cell cycle progression. We then assessed the expression levels of several DDR-related genes using RT-qPCR. The results revealed significantly higher expression levels of *ATM*, *ATR*, *BRCA1*, and *SMR7* in all four treatment groups compared to the NAT group (Fig. 4f). Additionally, *PARP2*, *MRE11,* and *RAD51* were significantly induced in the HAT 48h group, indicating that DDR might be activated in response to elevated temperatures, especially under HAT conditions (Fig. 4f).

To evaluate the impact of elevated temperatures on cell cycle-regulatory genes, we analyzed the expression levels of key markers associated with cell cycle progression. These included *CYCB1;2* and *KN*, which are specifically expressed during the G2/M phase transition, *TSO2* and *H3.1* for the S phase, and *CYCD3;3* for the G1 phase. Compared to the NAT group, *CYCB1;2* and *KN* expression was significantly reduced in both the HAT 2 h and HAT 48 h groups, whereas *CYCB1;2* levels increased under WAT conditions at both 2 h and 48 h (Fig. 4g). *H3.1* and *TSO2* exhibited decreased expression in the HAT 48 h group, but showed slight increases under WAT conditions. *CYCD3;3* expression increased in both the WAT 2 h and 48 h groups, and in the HAT 48 h group (Fig. 4g). Collectively, these results suggest that the DDR triggered by elevated temperatures leads to alterations in the expression of cell cycle-related genes in the root tips.

### Alternative Splicing in Response to Elevated Temperatures

**Fig. 5:**
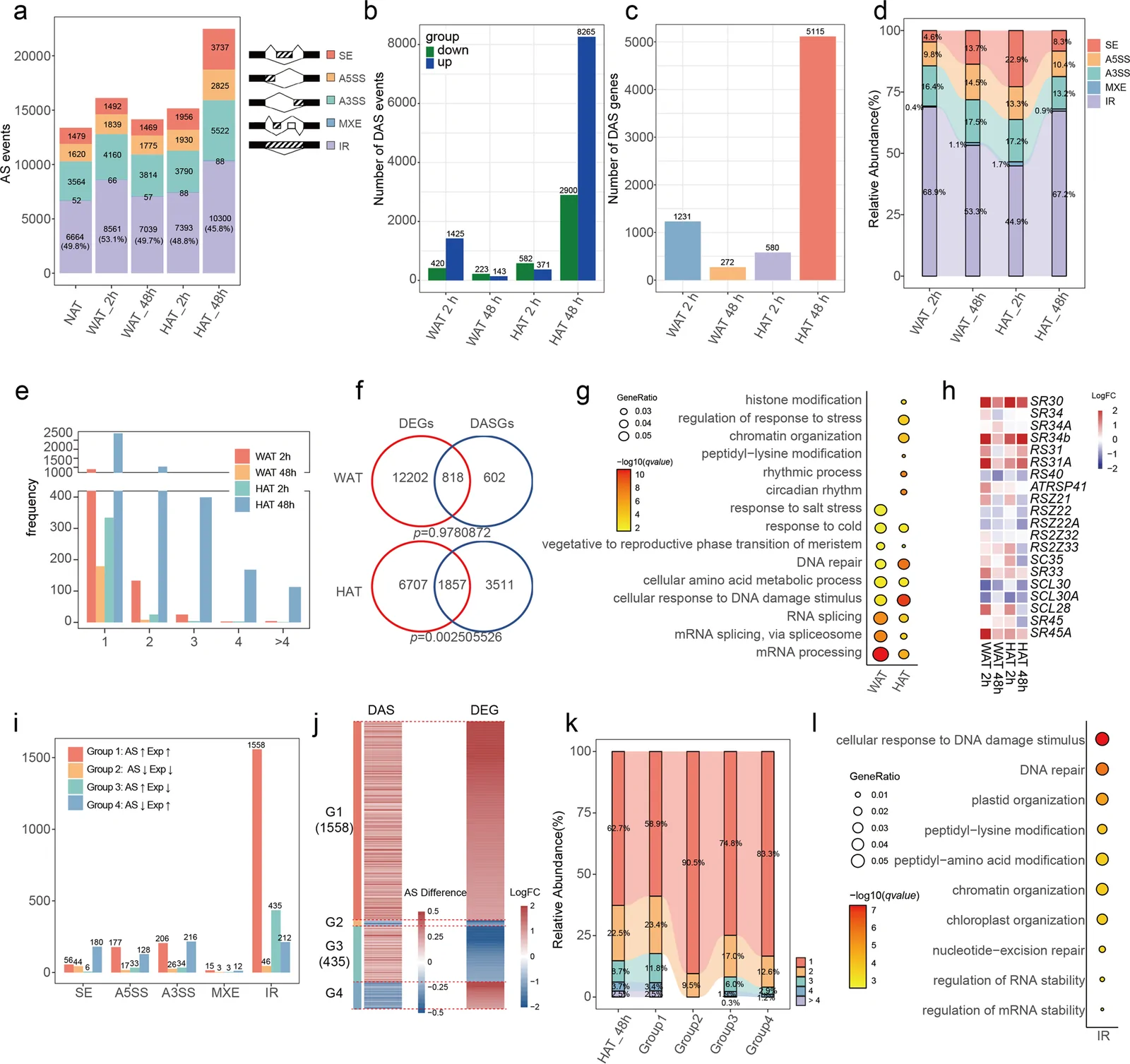
AS events in response to elevated temperatures. **a** Bar plot showing the number of AS events of the five main AS patterns under different temperature conditions. **b** Number of up-regulated and down-regulated differential AS (DAS) events after 2 or 48 h of WAT or HAT treatment. **c** Number of DASGs after 2 or 48 h of WAT or HAT treatment. **d** Bar plot showing the proportion of different categories of DAS events under the corresponding elevated temperatures. **e** Bar plot showing the frequency of genes exhibiting multiple differential IR events under different temperatures. **f** Venn diagram showing the overlap of DEGs and DASGs in response to WAT and HAT treatments. The *p-value* (hypergeometric test) indicates the significance of the overlap. **g** Dot plot showing the top Gene Ontology (GO) terms enriched for the overlapping genes between DASGs and DEGs under HAT or WAT conditions. **h** Heatmap showing the expression of splicing factors in response to elevated temperatures. **i** Bar plot showing the number of AS events across the five AS types in each overlapping DASG and DEG group under 48 h of HAT stress. **j** Heatmaps showing the splicing differences and expression changes of the overlapping genes with IR events from i. AS differences were calculated as (IncLevel HAT - IncLevel WAT). G1 (both AS and expression upregulated), G2 (both downregulated), G3 (AS upregulated and expression downregulated), and G4 (AS downregulated and expression upregulated). **k** Bar plot showing the frequency of G1 genes with multiple IR events. **l** Dot plot showing the top GO terms enriched for G1 genes associated with IR events

To investigate the common and distinct effects of varying levels of heat stress on mRNA AS processes, we conducted a comprehensive analysis of AS events under different temperature conditions. A total of 13,379, 16,118, 14,154, 15,157, and 22,472 AS events were identified in NAT, WAT 2 h, WAT 48 h, HAT 2 h, and HAT 48 h, respectively (Fig. 5a). These AS events were classified into five categories: intron retention (IR), skipped exon (SE), mutually exclusive exons (MXE), alternative 5’ splice site (A5SS), and alternative 3’ splice site (A3SS). Among these, IR events accounted for the largest proportion in all five samples representing 49.8%, 53.1%, 49.7%, 48.8%, and 45.8% of the total AS in NAT, WAT 2 h, WAT 48 h, HAT 2 h, and HAT 48 h, respectively (Fig. 5a). Given the predominance of IR among AS events, seven randomly chosen IR-type events from RNA-seq data were validated by RT-PCR. The differentially spliced patterns for all seven IR events before and after heat exposure were consistent between the RT-PCR and the transcriptomic data (Fig. S7), confirming the reliability of our analysis.

Compared to NAT, we identified 1845 (1425 upregulated and 420 downregulated), 366 (143 upregulated and 223 downregulated), 953 (371 upregulated and 582 downregulated), and 11,165 (8265 upregulated and 2900 downregulated) differential alternative splicing (DAS) events in WAT 2 h, WAT 48 h, HAT 2 h, and HAT 48 h, respectively (Fig. 5b). The highest number of DAS events (11,165) was observed under the 48 h HAT conditions, highlighting the significant impact of prolonged heat stress on AS. These DAS events were associated with 1231, 272, 580, and 5115 differentially spliced genes (DASGs) in WAT 2 h, WAT 48 h, HAT 2 h, and HAT 48 h, respectively (Fig. 5c), indicating that heat stress significantly alters AS patterns in these genes.

Among the DAS events induced by heat stress, the proportion of IR events markedly increased in WAT 48h and HAT 48 hsamples, rising from 49.7% to 53.3% in WAT 48 hand from 45.8% to 67.2% in HAT 48 h (Fig. 5d). Notably, the frequency of genes exhibiting multiple differential IR events also significantly increased in the HAT 48 h group (Fig. 5e), highlighting the dominance of IR during long-term heat stress.

### Comparative Analysis of DEGs and DASGs

To explore the relationship between AS and transcriptional regulation in response to elevated temperatures, we compared genes exhibiting differential alternative splicing (DASGs) with those showing differential gene expression (DEGs). Overlaps were observed between DASGs and DEGs, with 818 overlapping genes identified in WAT (including WAT 2 h and 48 h) and 1857 overlapping genes identified in HAT (including HAT 2 h and 48 h) (Fig. 5f), suggesting that both the transcriptional levels and splicing patterns of these genes are influenced by heat.

Functional enrichment analysis of these overlapping DEGs and DASGs revealed common enrichment in pathways related to mRNA processing, DNA repair, and RNA splicing, suggesting that both WAT and HAT conditions affect these processes. However, distinct pathways were enriched under different stress conditions: WAT specifically stimulated pathways associated with vegetative-to-reproductive phase transition of meristem, whereas HAT uniquely enriched pathways related to histone modification, chromatin organization, rhythmic processes, and DNA damage, particularly under the HAT 48 h conditions (Fig. 5g). These findings highlight the functional specificity of different heat stress levels in affecting plant growth.

Notably, genes encoding splicing factors such as *SR30*, *SR34b*, and *SR31A* showed increased expression accompanied by altered splicing patterns in response to elevated temperatures (Fig. 5h; Table S16). Additionally, genes involved in DNA repair (e.g., *KU80*, *KU70*, *XPB1*, and *XPB2*) and histone modification (*MRG1* and *MRG2*) exhibited significant AS changes under HAT conditions (Table S16-S17), suggesting that post-transcriptional regulation is activated in both the DNA damage response and histone modification pathways. To validate these findings, three IR events in DNA repair genes were randomly selected for verification via RT-PCR, and the results were consistent with the bioinformatic analysis (Fig. S8). These results collectively reveal that AS may serve as an additional regulatory layer in plant responses to heat, complementing transcriptional adjustments.

To further assess the association between IR events and the overlap of DAS and DEGs, we analyzed the proportion of IR events and the frequency of genes exhibiting multiple differential IR events among the 1556 overlapping genes under the HAT 48 h conditions (Fig. 5i, k). These genes were associated with 3407 AS events, of which 2251 (66.1%) were IR events (Fig. 5i). Based on the trends in AS changes and gene expression levels, the overlapping genes were categorized into four groups: G1 (both AS and expression up-regulated), G2 (both down-regulated), G3 (AS up-regulated and expression down-regulated), and G4 (AS down-regulated and expression up-regulated). Notably, the majority of IR events (1558 out of 2251, 67.2%) were classified in G1 (Fig. 5i and j). Furthermore, G1 genes demonstrated a significantly higher frequency of multiple differential IR events under heat stress. Specifically, the proportion of genes with only one IR event decreased from 62.7% to 58.9%, while the proportions of genes with two and three IR events increased from 22.5% to 23.4% and 8.7% to11.9%, respectively (Fig. 5k). Functional enrichment analysis revealed that these G1 genes were predominantly associated with pathways related to cellular response to DNA damage stimulus, DNA repair, plastid organization, and chromatin organization. Collectively, these findings suggest that prolonged heat stress not only increases the frequency of IR events but also drives a coordinated upregulation of gene expression in most IR-associated genes, highlighting a critical mechanism in plant adaptation to stress.

### Conserved Activation of DDR and Altered Cell Cycle Progression in Response to Elevated Temperatures in Tobacco and Tomato

**Fig. 6:**
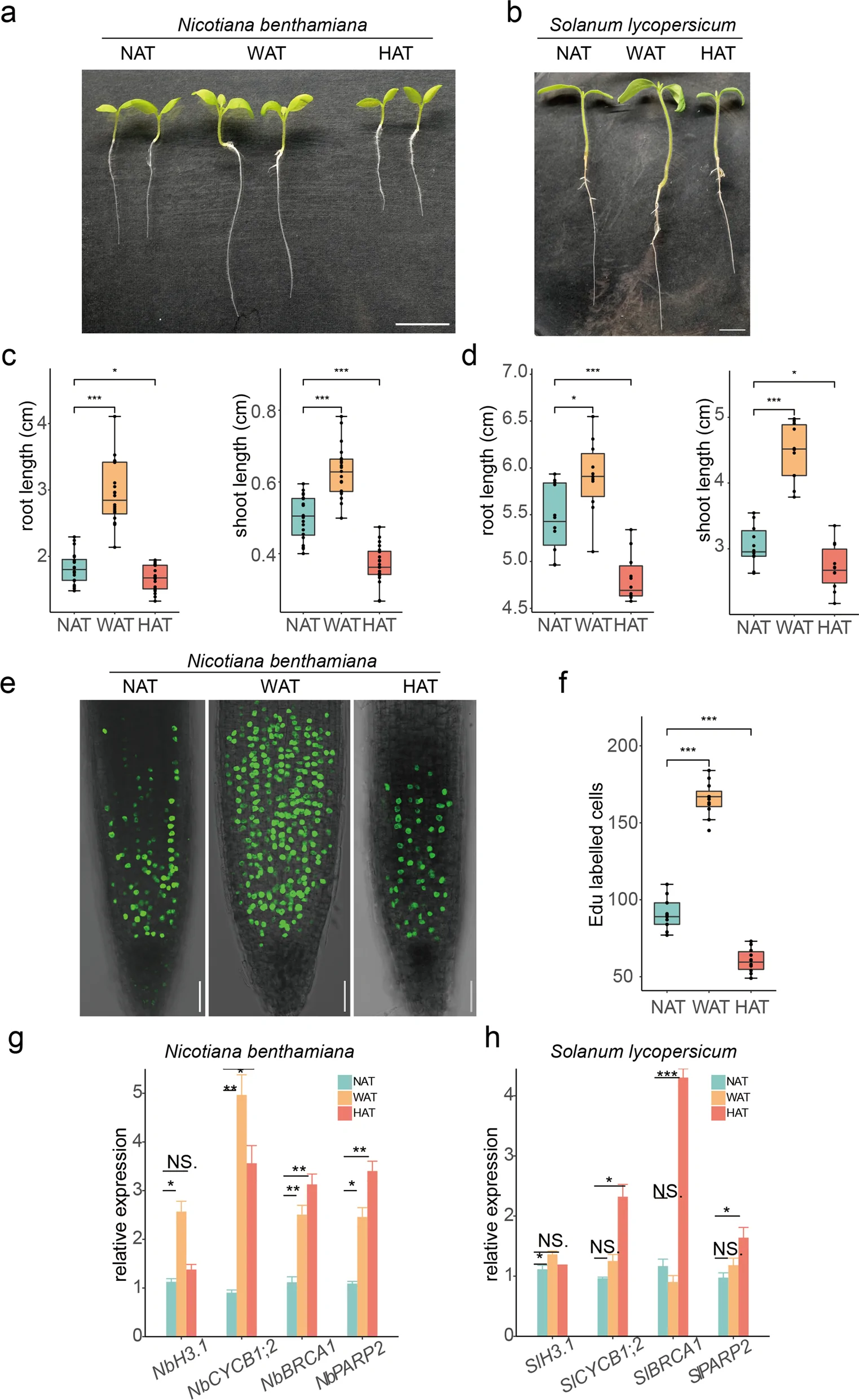
Elevated Temperatures Also Alter Cell Cycle Progression in Tobacco and Tomato. **a-d** phenotypes of tobacco and tomato in response to elevated temperatures. Tobacco seedlings were grown for four days on MS plates at 22 °C and then kept either at 28 °C or 37 °C for an additional three days. Tomato seedlings were grown for four days at 22 °C and then kept at 28–37 °C for additional three days. Bar plots show root length and shoot length for tobacco and tomato seedlings. **e** S phase entry of mitotic cells in tobacco roots were visualized by EdU staining. **f** Counting of EdU labeled cells in root meristem. **g-h** RT-qPCR analysis of cell cycle-related genes in seedlings in response to elevated temperatures. Statistical analysis was performed using Student’s *t*-test: *, *p* < 0.05; **, *p* < 0.01; ***, *p* < 0.001, ns, not significant

To further investigate the conservation of molecular mechanisms underlying plant responses to varying levels of thermal stress, we subjected tobacco (*Nicotiana benthamiana*) and tomato (*Solanum lycopersicum* 'Ailsa Craig') seedlings to WAT and HAT conditions compared to NAT, and examined their growth status and molecular responses. After growing at NAT for four days, the seedlings were transferred to WAT or HAT for an additional three days. Similar to *Arabidopsis*, both tobacco and tomato exhibited enhanced root and shoot growth under WAT conditions and inhibited growth under HAT conditions (Fig. 6a, d), suggesting a conserved regulatory mechanism in response to elevated temperatures. To further investigate whether cell cycle progression was also altered in tobacco, we used EdU labeling to mark cells entering the S phase. Consistent with *Arabidopsis* results, the number of EdU-labeled cells significantly increased under WAT and decreased under HAT (Fig. 6e, f), indicating enhanced cell division under WAT and inhibition under HAT. Additionally, we performed RT-qPCR to analyze the expression of homologs of *H3.1*, *CYCB1;2*, *BRCA1,* and *PARP2* in tobacco and tomato. In both tobacco and tomato, the transcript levels of homologs of H3.1 were elevated under WAT conditions, while the homologs of *BRCA1* and *PARP2* showed increased expression in response to HAT conditions. Interestingly, the *CYCB1;2* homologs in tobacco and tomato exhibited higher transcript levels under both WAT and HAT conditions (Fig. 6g, h). This might suggest that the function of these genes differs from those in *Arabidopsis*. Overall, our findings indicate that in response to elevated temperatures, the DDR mechanism may also be activated in both tobacco and tomato, leading to altered cell cycle progression. Taken together, our data indicate that the DDR mechanism might be conserved across plant species and could play a role in their responses to elevated temperatures.

## Discussion

Elevated temperatures are a critical environmental factor significantly influencing plant growth, development, and crop yield. As ambient temperatures rise from room temperature (approximately 22 °C) to levels that induce thermomorphogenesis (around 28 °C), plants experience a transition from regular growth to thermomorphogenic growth, often accompanied by accelerated growth rates. However, when temperatures reach heat stress-inducing levels (around 37 °C), plants experience growth inhibition and stress-induced physiological disruptions. Our study aimed to comprehensively explore the molecular mechanisms underpinning plant responses to varying thermal conditions, specifically warm ambient temperature (WAT, 28 °C) and high ambient temperature (HAT, 37 °C). By investigating both short-term and long-term responses to these temperature conditions, we sought to uncover how plants balance growth and stress responses when subjected to different levels of thermal stress.

### Plants Utilize Specific Transcription Factors Depending on the Intensity and Duration of Thermal Stress

Interestingly, although thermomorphogenesis and heat stress responses are often considered separate processes, our results suggest potential overlaps, particularly in pathways related to the DNA damage response (DDR) and cell cycle regulation (Fig. 2). Given that plants often encounter fluctuating temperatures, transitioning from WAT to HAT conditions, the intersection of these processes warrants further investigation. Our study provides evidence that DNA repair and cell cycle regulation are activated in both WAT and HAT conditions, but with distinct temporal patterns. It is important to note that the comparison between NAT and 2 h WAT/HAT conditions may capture not only heat-responsive transcriptional changes but also circadian and photoperiod-dependent fluctuations.

We observed that the number of differentially expressed genes (DEGs) was significantly higher under short-term (2 h) WAT conditions than under short-term HAT conditions, and also exceeded the number of DEGs observed under both long-term (48 h) WAT and HAT conditions (Fig. 2). In contrast, short-term HAT exposure elicited fewer DNA damage response (DDR) responses and minimal gene expression changes, whereas short-term WAT triggered higher DDR activity and substantial transcriptional alterations. These findings suggest that short-term WAT induces an immediate and intense transcriptional response, particularly in activating DDR mechanisms, while the upregulation of DDR genes under short-term HAT is delayed.

Conversely, prolonged HAT exposure (48 h) appears to induce more extensive gene expression changes than prolonged WAT exposure (Fig. 2a). These findings are consistent with previous studies in alfalfa, which reported higher DEG counts (5482 and 4341) after 72 h of HAT exposure (35 °C and 40 °C) compared to 72 h of WAT exposure (3400, 30 °C) (Zhou et al. 2024b).

Furthermore, prolonged HAT exposure (48 h) results in more severe DNA damage accumulation (Fig. 3d, e). Moreover, although long-term HAT exposure elevates DDR-related gene expression relative to short-term HAT, the overall expression levels remain lower than those induced by short-term WAT treatment (Fig. 3c). This delayed induction of DDR genes suggests impaired timely repair of DNA damage, leading to a greater accumulation of DNA damage under HAT conditions compared to WAT (Fig. 3d).

In addition to DNA repair, cell cycle regulation exhibited temperature-dependent differences. While WAT promoted cell division and growth by facilitating progression into the S phase, prolonged HAT treatment induced a G2/M arrest, inhibiting cell cycle progression and growth. This arrest may not only result from the accumulation of DNA damage but also from potential impairment of key DDR proteins that regulate the G2/M checkpoint. In particular, DDR-related proteins play critical roles in monitoring DNA damage and ensuring proper checkpoint activation (Pedroza-Garcia et al. 2022). Under HAT conditions, the expression of these genes is delayed (Fig. 3c), which caused their proteins to fail to function immediately and properly, subsequently contributing to the observed G2/M arrest (Fig. 4c, e, g). This suggests that, beyond the accumulation of DNA damage, there could be a failure in the DNA damage sensing and repair machinery, accelerating cell cycle arrest in HAT conditions, particularly under long-term HAT conditions. Together, these findings suggest that plants can modulate their cell cycle response depending on the severity and duration of heat stress, with strategies for growth promotion under moderate heat and stress adaptation under extreme conditions.

A critical insight from our study is the role of DDR activation and cell cycle dynamics as a shared but modifiable pathway in response to WAT and HAT. Previous research has shown that various stresses, including reactive oxygen species (ROS) accumulation, UV radiation, and salinity, can trigger DDR and alter cell cycle progression (Cui et al. 2017; Pedroza-Garcia et al. 2022; Yi et al. 2014). Our findings are in line with these studies, albeit with distinct outcomes depending on the temperature intensity. Interestingly, the natural variation of ICA1 alleles has been reported to modulate cell cycle progression, enabling plants to adapt to fluctuating environmental conditions (Zhu et al. 2015). These insights into the impact of elevated temperature on DDR and cell cycle regulation emphasize the temperature-dependent balance between growth promotion and stress adaptation.

Another significant aspect of our study is the conservation of these mechanisms across plant species. We observed similar heat-induced cell cycle changes in *Arabidopsis*, tobacco, and tomato (Fig. 6). This conservation strengthens our understanding of plant resilience to environmental fluctuations and offers valuable insights for crop improvement in the face of climate change. The identification of conserved pathways and genes involved in temperature response provides a foundation for breeding heat-tolerant crops that can maintain productivity under variable climatic conditions.

Our work also contributes to advancing high-throughput phenotyping approaches for heat tolerance. By identifying key genes involved in DNA damage repair (DDR), cell cycle regulation, and stress response, we provide molecular markers that could be used to screen plant populations for heat tolerance. For example, genes such as *XPD*, *RAD9* (DDR), *KRP7*, *CDKD-2*, and *CDKA-1* (cell cycle regulation), as well as *PSBP1*, *LHCA5*, and *PSBO1* (photosynthesis), were specifically upregulated under 37 ℃ heat stress. Conversely, genes such as *MCM2*, *MCM5*, and *MYB3R4,* were up-regulated at 28 ℃ but downregulated at 37 ℃. These differential expression patterns can serve as molecular markers in high-throughput phenotyping platforms, enabling rapid screening for heat tolerance in breeding programs. Furthermore, we observed that specific changes in root and hypocotyl growth patterns under heat stress are associated with distinct gene expression profiles, such as those of *EXPB1*, *EXPA15*, and *PCNA*. These correlations provide a foundation for developing automated phenotyping tools that utilize advanced imaging technologies, such as hyperspectral imaging and thermal cameras, to non-invasively detect phenotypic changes linked to the expression of heat-responsive genes. Integrating these technologies into high-throughput phenotyping pipelines could greatly enhance the ability to monitor plant responses to heat stress in real-time, across multiple genotypes.

### Alternative Splicing Events as a Regulatory Layer in Heat Stress Adaptation

One of the most striking findings of our study is the significant role of AS, particularly IR events, in mediating plant responses to prolonged heat stress. Our comprehensive analysis reveals that genes associated with DNA repair, chromatin organization, and plastid organization undergo pronounced AS changes, with a predominant increase in IR events. Furthermore, we identified a clear correlation between upregulated gene expression and increased IR events, especially in genes involved in DNA repair and cellular response to stress. These results suggest that AS, and specifically IR, may serve as an essential mechanism for fine-tuning gene expression under heat stress, enabling plants to modulate their responses at the post-transcriptional level.

We identified significant correlations between gene expression changes and AS events under heat stress (Fig. 5), particularly in genes involved in DNA repair (*ATM*,* INO80*, and *RAD50*). AS is a prevalent phenomenon in both animals and plants (Marquez et al. 2012; Pan et al. 2008), and stress-induced AS provides an additional layer of gene regulation (Ding et al. 2014; Li et al. 2020; Ling et al. 2021). We found that both warm ambient temperature (WAT) and high ambient temperature (HAT) induce significant alterations in AS patterns, with distinct responses observed between short-term and long-term heat stress conditions.

Notably, we observed that prolonged heat stress is associated with increased intron retention (IR) events, which correlate with upregulated gene expression levels. This also coincides with an elevated frequency of IR in mature mRNA transcripts (Fig. 5j). These results suggest that AS, particularly IR, may influence gene expression outcomes, potentially leading to the generation of alternative protein isoforms, triggering transcript degradation via nonsense-mediated decay (NMD), or the retention of transcripts in the nucleus as detained introns. Despite extensive studies on the relationship between DNA damage response (DDR) and AS in animal systems, much less is known about this interaction in plants (Shkreta and Chabot 2015). However, several key DNA repair genes have been reported to undergo AS, underscoring its significance in DDR within plant systems (Nimeth et al. 2020). Our findings indicate that the AS patterns of DNA repair genes exhibit substantial changes in response to elevated temperatures (Table S16), suggesting that AS may play a regulatory role in DDR related to heat stress in plants. The sequencing depth used in this study (20 million reads per replicate) is sufficient to capture major alternative splicing events (Hong et al. 2023; Jabre et al. 2021; Zhou et al. 2024a), but it may not fully resolve low-abundance isoforms. While our findings (Fig. 5a, g, h) align with previously reported heat stress-induced splicing patterns (Ling et al. 2021), deeper sequencing or long-read approaches could enhance the resolution of rare AS events and provide a more comprehensive transcriptome profile. Incorporating these findings into high-throughput phenotyping frameworks could significantly improve the precision of identifying plants with beneficial AS patterns, contributing to the development of heat-tolerant crops.

## Conclusion

Our study demonstrates the intricate and dynamic regulation of gene expression, DDR activation, and cell cycle progression in response to elevated temperatures. These mechanisms are conserved across different temperature conditions and plant species, offering new insights into plant adaptation and resilience to thermal stress. Moreover, the integration of AS into heat stress responses provides a novel regulatory layer that could be leveraged for crop improvement. Future studies could focus on how various temperature regimes, including nighttime temperatures, influence plant growth and stress responses, further enhancing our understanding of plant adaptation to changing climates.

## Electronic Supplementary Material

Below is the link to the electronic supplementary material.

Supplementary Material 1

Supplementary Material 2

## Data Availability

The sequencing data generated in this study have been deposited in NCBI Sequence Read Archive (SRA) database with the accession number: PRJNA1158535. The datasets generated for this study are available upon request from the corresponding authors.

## Abbreviations

Arabidopsis: *Arabidopsis thaliana*
DDR: DNA damage response
DEGs: Differentially expressed genes
DASGs: Differentially alternatively spliced genes
HAT: High ambient temperature
HSF: Heat shock transcription factor
HSP: Heat shock protein
NAT: Normal ambient temperature
PIF4: Phytochrome interacting factor 4
WAT: Warm ambient temperature
ROS: Reactive oxygen species
AS: Alternative splicing
IR: Intron retention
