# Supplementary material for: Comprehensive Analysis of RNA Expression and Isoform Diversity Reveals Molecular Mechanisms in Plant Responses to Diverse Thermal Conditions
Source: Phenomics. 2026 Jun 24;6(4):467–84. doi: 10.1007/s43657-025-00254-5 (PMC13713942; doi:10.1007/s43657-025-00254-5)
Supplement: Supplementary file 1 [file 43657_2025_254_MOESM2_ESM.docx]

**Fig. S1 Verification of RNA-seq results.**

**a** Principal component analysis (PCA) for gene expression data of seedlings subjected to different temperature regimes and treatment durations. **b** Hierarchical clustering analysis for four treatment conditions. **c** Scatter plots showing the correlation between RNA-seq and RT-qPCR results across all groups. The x-axis represents the Log2 fold change (Log2FC) relative to NAT as the control, while the y-axis indicates normalized qPCR expression. Most genes displayed strong consistency between RNA-seq and RT-qPCR data, with Spearman correlation coefficients (R²) value exceeding 0.8. At least seven genes were validated per group. **d** Expression levels of the qPCR internal control gene *AT1G13320* as measured by RNA-seq. **e, f** Venn diagrams showing the overlap among different WAT or HAT transcriptomes. DEGs for HAT and WAT are derived from this study.

**Fig. S2** **Expression profile of common DEGs across all four treatments.**

**a** Clustering analysis of 625 common DEGs across all groups based on their expression patterns (Log2FC, log2 fold change). **b** Boxplot illustrating the expression levels of heat shock protein (HSP) and heat shock factor (HSF) genes. **c** Heatmap showing the expression profiles of selected HSP/HSF genes, salicylic acid (SA)-related genes, and other selected genes of interest (Log2FC, log2 fold change).

**Fig. S3** **Comparison of long-term effects of WAT and HAT on Arabidopsis seedlings.**

**a** Venn diagram showing the overlaps of GO terms enriched in the up-regulated genes under WAT and HAT conditions. **b-d** Dot plots illustrating the GO terms enriched in long-term treatments groups for WAT and HAT up-regulated genes. **e** Venn diagram showing the overlap of GO terms enriched in down-regulated genes under WAT and HAT conditions. **f-h** Dot plots illustrating the GO terms enriched in long-term treatments groups for WAT and HAT down-regulated genes.

**Fig. S4 Comparison of short-term effects of WAT and HAT on Arabidopsis seedlings.**

**a** Venn diagram displaying the overlaps of GO terms enriched in up-regulated genes under WAT and HAT conditions. **b-d** Dot plots illustrating the GO terms enriched in short-term treatment groups for WAT and HAT up-regulated genes. **e** Venn diagram displaying the overlaps of GO terms enriched in down-regulated genes under WAT and HAT conditions. **f-h** Dot plots illustrating the GO terms enriched in short-term treatment groups for WAT and HAT down-regulated genes.

**Fig. S5 Induction of genes involved in different DNA repair mechanisms by WAT and HAT.**

**a-c** Boxplots showing the expression levels of DEGs involved in Homologous recombination repair, nucleotide excision repair, and mismatch excision repair.

**Fig. S6 Alteration of auxin concentration in response to elevated temperatures.**

**a** GUS staining of *DR5::GUS* in root tips under NAT, WAT, and HAT conditions. **b** Quantification of *DR5::GUS* activity in root tips (n>10). Scale bar = 50 µm. Statistical analysis performed using Student’s t-test: *, *p*< 0.05; **, *p* < 0.01: ***, *p* < 0.001; Ns., not significant.

**Fig. S7 Validation of IR events identified by RNA-seq. a-h** Gene structures of intron retention (IR) event-related genes are shown at the top. Exons are shown as black boxes, UTR regions as white boxes, introns without IR as black lines, and retained introns as red lines. Red boxes highlight the positions of the retained introns. Arrows indicate primer locations used for RT-PCR validation. The IR events identified by RNA-seq were visualized using the Integrative Genomics Viewer (IGV) and are shown on the left side of each panel. Wiggle plots display the normalized read coverage for the IR events on a logarithmic scale (log2). Reads were normalized using RPKM (Reads Per Kilobase of transcript, per Million mapped reads). RT-PCR validation results are shown on the right side of each panel. The experiments were performed with three independent biological replicates, yielding consistent results. Red arrows indicate the positions of intron-retained transcripts, while green arrows indicate the positions of intron-spliced transcripts. Actin was used as an internal control for RT-PCR, as well as negative control for AS events. Detailed information on the differences in AS levels detected by RNA-seq is provided in Table S16, includes information on the specific genes exhibiting differential splicing, the type of splicing events (e.g., IR, ES), and the statistical significance of these changes.

**Fig. S8 Validation of Long-term heat stress induced changes in the IR events of DNA repair related genes. a-d** Gene structures of intron retention (IR) event-related genes are shown on the top. Exons are shown as black boxes, UTR regions as white boxes, introns without IR as black lines, and retained introns as red lines. Red boxes highlight the positions of the retained introns. Arrows indicate primer locations used for RT-PCR validation. The IR events identified by RNA-seq were visualized using the Integrative Genomics Viewer (IGV) and are shown on the left side of each panel. Wiggle plots display the normalized read coverage for the IR events on a logarithmic scale (log2). Reads were normalized using RPKM (Reads Per Kilobase of transcript, per Million mapped reads). RT-PCR validation results are shown on the right side of each panel. The experiments were performed with three independent biological replicates, yielding consistent results. Red arrows indicate the positions of intron-retained transcripts, while green arrows indicate the positions of intron-spliced transcripts. Actin was used as an internal control for RT-PCR, as well as negative control for AS events. Detailed information on the differences in AS levels detected by RNA-seq is provided in Table S16.
